# Characterization of Dentine to Assess Bond Strength of Dental Composites

**DOI:** 10.3390/ma8052110

**Published:** 2015-04-24

**Authors:** Saad Liaqat, Anas Aljabo, Muhammad Adnan Khan, Hesham Ben Nuba, Laurent Bozec, Paul Ashley, Anne Young

**Affiliations:** 1 Division of Biomaterials and Tissue Engineering, UCL Eastman Dental Institute, 256 Gray’s Inn Road, London WC1X 8LD, UK; E-Mails: saad.liaqat.11@ucl.ac.uk (S.L.); anas.aljabo.10@ucl.ac.uk (A.A.); adnan.khan.11@ucl.ac.uk (M.A.K.); hesham.nuba.09@ucl.ac.uk (H.B.N.); l.bozec@ucl.ac.uk (L.B.); 2 Department of Paediatric Dentistry, UCL Eastman Dental Institute, 256 Gray’s Inn Road, London WC1X 8LD, UK; E-Mail: p.ashley@ucl.ac.uk

**Keywords:** dental composite, dentine, 4-META, self-adhesive, hydroxyapatite, collagen, micro-gap

## Abstract

This study was performed to develop alternating dentine adhesion models that could help in the evaluation of a self-bonding dental composite. For this purpose dentine from human and ivory was characterized chemically and microscopically before and after acid etching using Raman and SEM. Mechanical properties of dentine were determined using 3 point bend test. Composite bonding to dentine, with and without use of acid pre-treatment and/or the adhesive, were assessed using a shear bond test. Furthermore, micro gap formation after restoration of 3 mm diameter cavities in dentine was assessed by SEM. Initial hydroxyapatite level in ivory was half that in human dentine. Surface hydroxyapatites decreased by approximately half with every 23 s of acid etch. The human dentine strength (56 MPa) was approximately double that of ivory, while the modulus was almost comparable to that of ivory. With adhesive use, average shear bond strengths were 30 and 26 MPa with and without acid etching. With no adhesive, average bond strength was 6 MPa for conventional composites. This, however, increased to 14 MPa with a commercial flowable “self–bonding” composite or upon addition of low levels of an acidic monomer to the experimental composite. The acidic monomer additionally reduced micro-gap formation with the experimental composite. Improved bonding and mechanical properties should reduce composite failures due to recurrent caries or fracture respectively.

## 1. Introduction

Dental caries, otherwise known as tooth decay, is one of the most prevalent chronic diseases of people worldwide [1]. It is caused by bacteria producing acid that enhances the solubility of hydroxyapatite in enamel and underlying dentine. Over time this process will lead to demineralization and cavitation [2]. Once the cavity is through to the dentine the infected areas must be removed and replaced by a filling material to restore tooth function and prevent continuing decay. Following the Minamata convention on mercury, phasing out of amalgam fillings will become likely. Dental composites will then be the main direct tooth restorative material. Composites have good esthetics, adhesion, and requires minimal cavity preparation. But on the other hand they are technically more difficult to place, and have higher failure rates due primarily to secondary caries beneath the restoration [3,4,5].

Conventionally, the most reliable composite bond has been achieved by first acid etching the dentine [6]. This provides a porous hydroxyapatite depleted surface collagen mesh and opens up aqueous fluid filled dentine tubules. Flowable hydrophilic dentine primers and adhesives can penetrate and upon polymerization physically interlock with both collagen and tubules [7]. The adhesives can additionally contain acidic monomers that can form ionic bonds with the dentine. Furthermore, the adhesive is able to chemically bond with the viscous hydrophobic composites. In an attempt to increase simplicity and reduce complexity, in the last decade, a major drive has been towards “single step” adhesives that may bond without etching. Their clinical success, however, has been variable. More recently, “flowable” composites, that have the potential to bond without etching or adhesive, have also been produced [8]. These flowable composites, however, can have high shrinkage enhancing micro-gap formation.

Predicting clinical success and optimization of new dental adhesives has been difficult. Many studies have used extracted human and bovine teeth to evaluate the adhesive strength of restorative materials. Human teeth, however, are difficult to obtain. Other problems include their small size, variability with age and disease level [9,10,11], infection hazard [12], and ethical issues [13]. Bovine teeth on the other hand are easy to obtain, but have same drawbacks like human teeth, which includes small size, variability, and cross infection. Alternative dentine models to quantify and provide better understanding of factors affecting bonding are therefore required. Confiscated ivory destined for destruction is available from customs but must be for academic purposes only. The relatively massive size of a single tusk, however, provides a potentially more reproducible substrate for academic research. The aim of this study was therefore to assess if ivory is a good replacement model for human dentine. The study additionally compares the dentine bonding of various composites with and without a single step adhesive and/or acid etching. The composites include conventional and flowable materials in addition to a new viscous composite with potential for self-bonding to dentine.

## 2. Results and Discussion

### 2.1. Results

#### 2.1.1. Raman and SEM of Dentine

**Figure 1:**
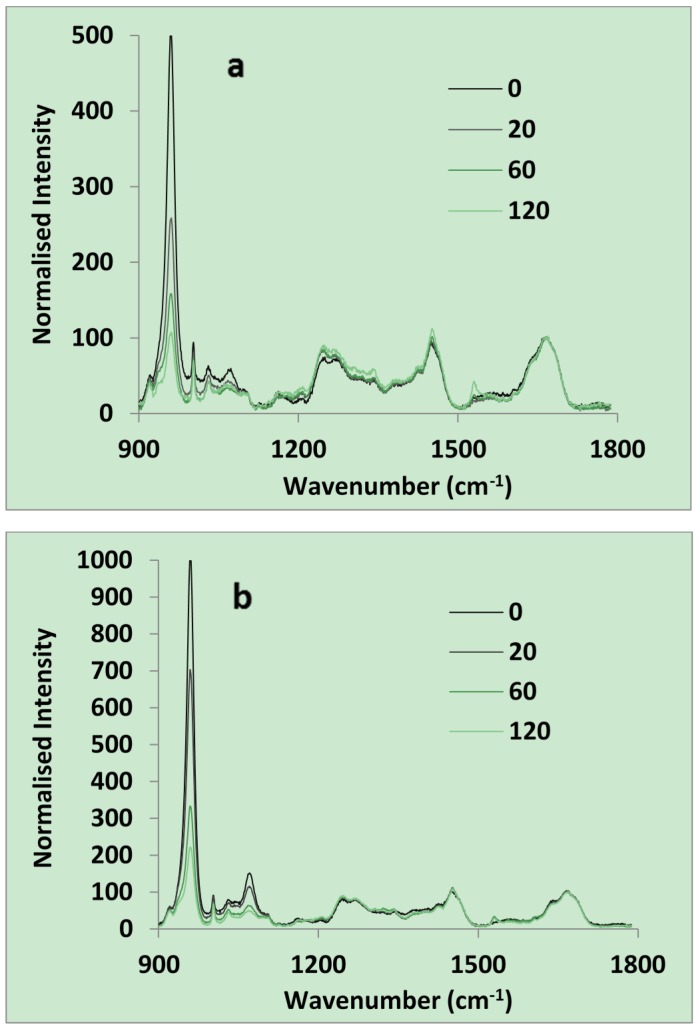

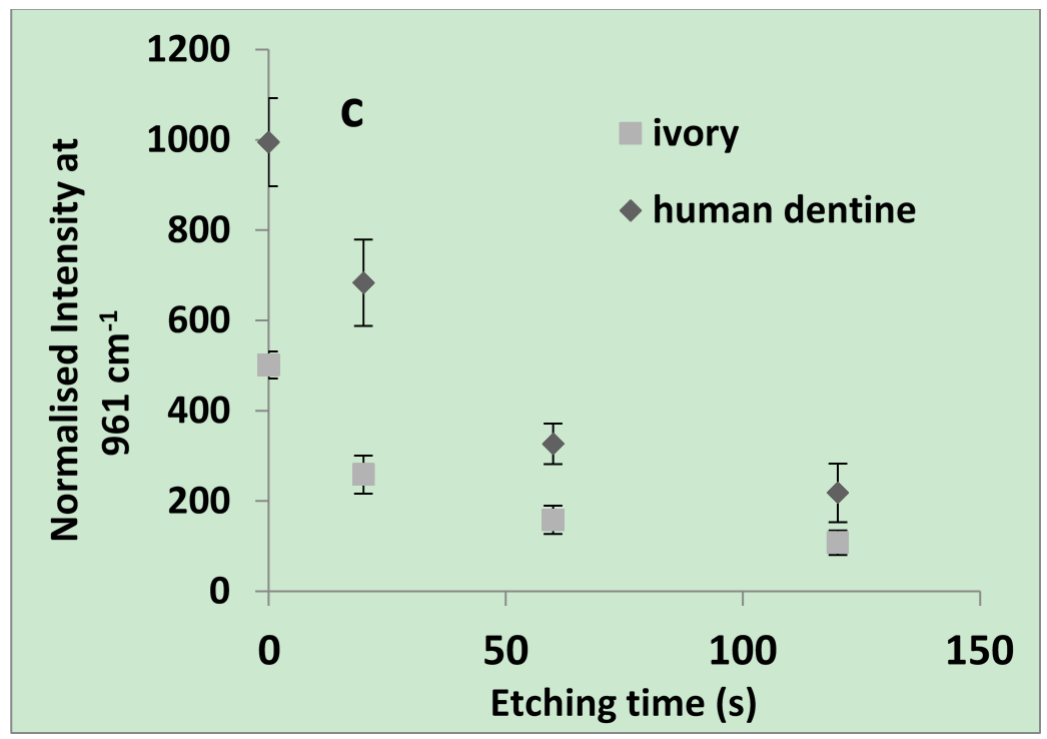
Chemical analysis of dentine using Raman spectroscopy. Raman spectra of (**a**) ivory; and (**b**) human dentine both after 0, 20, 60, or 120 s of acid etching; (**c**) Normalized intensity at 961 cm^−1^ is plotted against etching time in seconds (*n =* 5).

The normalized average spectra of both dentine were practically identical between 1200 and 1800 cm^−^^1^. This range included the 1670 cm^−1^ amide I, 1453 cm^−1^ amide II, and 1260 cm^−1^ amide III peaks due to collagen (Figure 1a,b). Below 1200 cm^−1^ both the hydroxyapatite (961 cm^−1^) and β- CO_3_^−2^ (1073 cm^−1^) peaks were more intense for the human dentine. After acid etching with 37% phosphoric acid for 20, 60 and 120 s both dentine hydroxyapatite and carbonate peaks declined (Figure 1c). The 20 s etched human dentine was almost identical to that of ivory except for having a much stronger carbonate peak.

Normalized intensity at 961 cm^−1^ for both dentine could be described well by an Equation (1) of the form:

(1) $$[\text{ ln}\left[\frac{I_{t}-I_{f}}{I_{0}-I_{f}}\right]=-0.03t]\;R^{2}\;>\;0.99$$

where *I* is intensity and subscripts *t*, *0* and *f* indicate times *t*, initial and final. This indicates the time for half maximum surface reaction is 23 s (−(ln (0.5))/0.03) for both dentine type. For ivory and human dentine *I_0_* values were 1000 and 500 and *I_f_* values 190 and 105 respectively.

**Figure 2:**
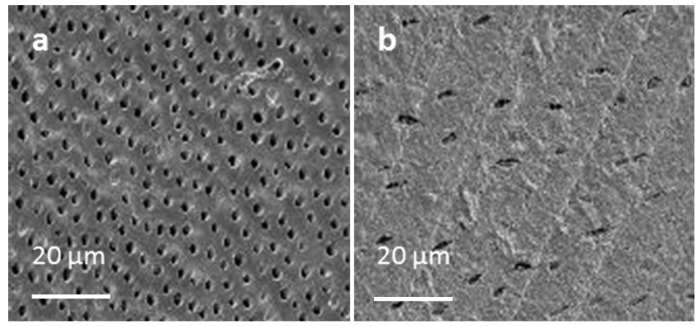
SEM images of (**a**) human dentinal tubules near the pulp; (**b**) tubules in ivory.

Example SEM images of acid etched human dentine and ivory are provided in Figure 2. With human dentine the dentine tubule density (number/mm^2^) increased progressively from approximately 15,000 near the crown to 30,000 near the pulp. With ivory the tubule density was ~10,000 mm^−2^.

#### 2.1.2. Flexural Properties

**Figure 3:**
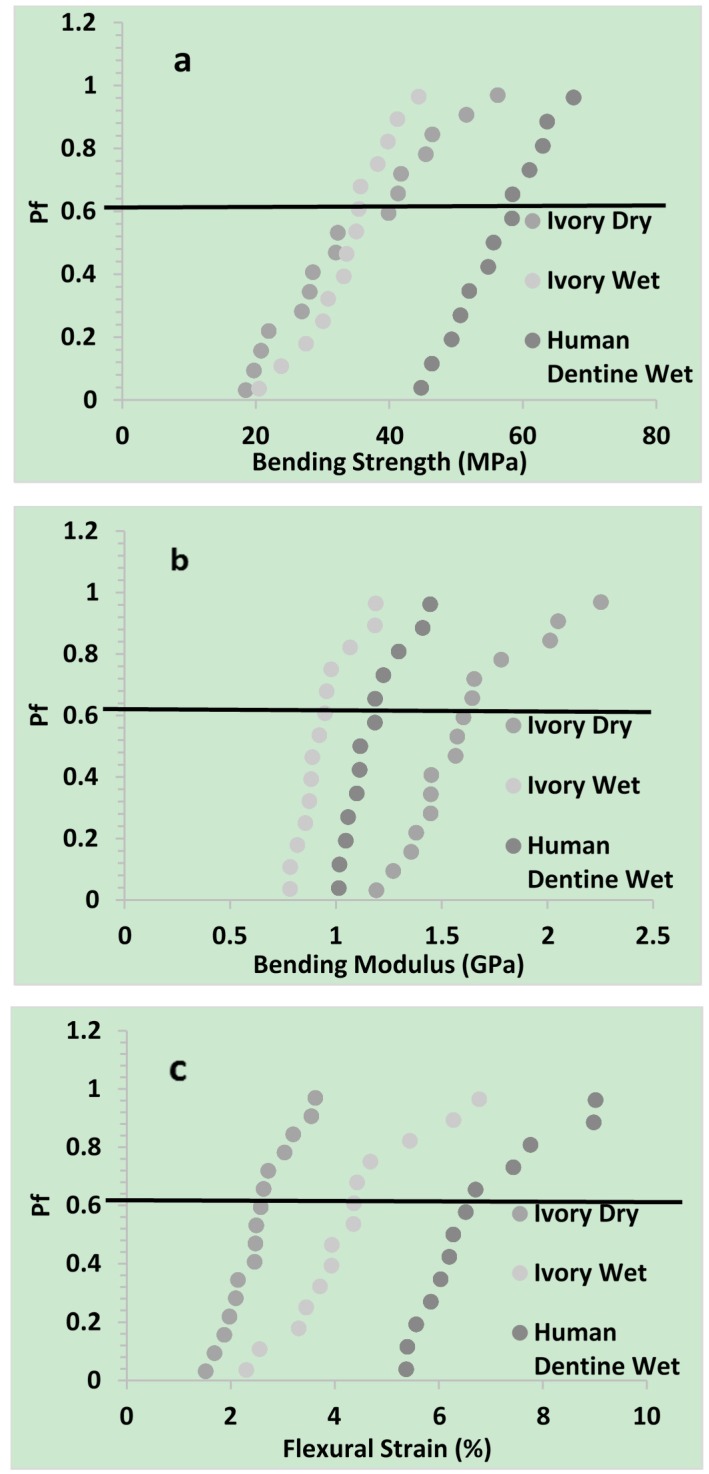
*P_f_* was plotted against (**a**) three point bend strength; (**b**) modulus; and (**c**) flexural strain of ivory (dry & wet) and human dentine (wet).

*P_f_* (Equation (5)) was plotted *vs.* bending strength, modulus, and flexural strain of dentine in Figure 3.

Mean results and Weibull parameters obtained by fitting Equation (6) are provided in Table 1. The high *R^2^* values indicated the Weibull distribution described strength variation particularly well but could also fit modulus and strain data. The Weibull strength scale parameter, σ_θ_, and mean strength were significantly higher for human dentine as compared to ivory dry and wet. With dry ivory, the reduced shape parameter, *m*, and greater 95% CI, indicated a broadened distribution of strengths compared with wet ivory or human dentine. Conversely, the Weibull scale parameter and mean modulus, increased in the order wet ivory < human wet dentine < dry ivory. Flexural strain increased in the order dry ivory < wet ivory < human wet dentine. Modulus and strain shape parameters, were not significantly affected by dentine type.

**Table 1:** Weibull scale *σ_θ_* and shape *m* parameters obtained upon fitting Equation (6) to three point strength, modulus, and flexural strain data.

Three point strength	Weibull parameter	Ivory dry	Ivory wet	Human dentine wet
Bending Strength (MPa)	*σ_θ_*	38	36	59
	*m*	3	6	8
	*R^2^*	0.97	0.99	0.99
	*Mean*	34	34	56
	*95% Cl*	6	4	4
Bending Modulus (GPa)	*σ_θ_*	1.7	1.0	1.2
	*m*	6	7	8
	*R^2^*	0.90	0.90	0.91
	*Mean*	1.6	0.9	1.2
	*95% Cl*	0.1	0.1	0.1
Flexural Strain (%)	*σ_θ_*	2.7	4.7	7.2
	*m*	4	4	5
	*R^2^*	0.96	0.96	0.90
	*Mean*	2.5	4.3	6.7
	*95% Cl*	0.3	0.7	0.7

#### 2.1.3. Shear Bond Strengths of Composites to Dentine

**Figure 4:**
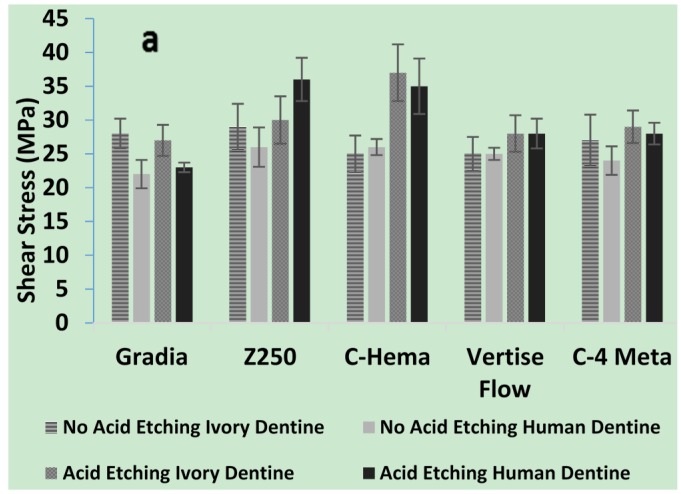

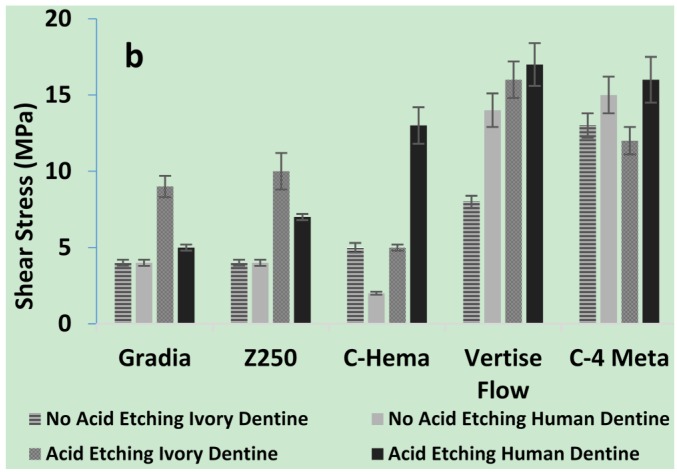
Shear bond strength of experimental and commercial composites, using dentine. (**a**) Shows the effect of ibond along with or without acid etching (20 s); (**b**) shows the effect of No ibond with or without acid etching (20 s) (*n =* 5).

From the combined results the biggest factor increasing bond strength was use of the adhesive ibond (average of 28 with *vs.* 9 MPa without). Acid etching increased average bond strengths both with (26 to 32 MPa) and without (7 to 12 MPa) use of adhesive (compare Figure 4a,b).

With ibond use, bond strengths with both dentine were almost comparable (Figure 4a). Without ibond, Gradia and Z250 bonded best to acid etched ivory whist the experimental C-HEMA composite bonded better to acid etched human dentine. Vertise flow had bond strengths above 10 MPa to all surfaces except un-etched ivory. The C-4META composite had bond strengths above 10 MPa for all dentine surfaces (Figure 4b).

#### 2.1.4. SEM of Composite/Dentine Interfaces

**Figure 5:**
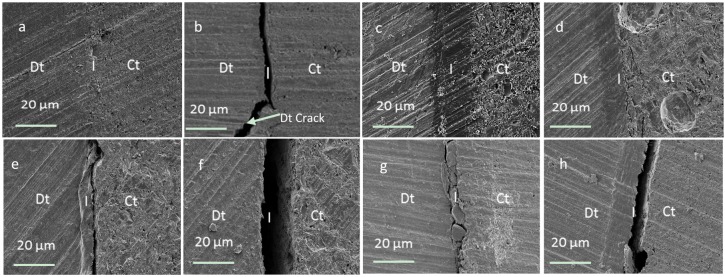
SEM images of interfaces between composites and dentine (**a**) ibond treated dentine with 4-META composite shows 3 different zones (dentine (Dt), interface (I), & composite (Ct)); (**b**) with ibond treated dentine and Z250 some interface and dentine cracking is observed; (**c**) acid and ibond treated dentine with 4-META composite has an ~20 micron thick interface layer; (**d**) Acid etched dentine with 4-META composite shows only minor cracking within the composite; (**e**) Un-treated dentine with 4-META composite has gaps of <2 micron whilst (**f**) un-treated dentine with HEMA composite has gaps >10 micron; (**g**) ibond treated dentine and Z250, failure at dentine interface can be seen; (**h**) Is acid and ibond treated dentine and Z250, the failure can be seen at dentine-ibond interface.

The SEM images of interfaces were comparable with ivory and human dentine. With the use of ibond adhesive and no acid etching a thin intact interface layer of <2 micron was typically observed between the dentine and most composites (e.g., Figure 5a). The only exception was with Z250. In this case, on one side of the restoration, some cracking of the dentine and/or interface was observed (Figure 5b). If the dentine was acid etched prior to ibond use this problem was overcome. Additionally a thicker adhesive/dentine hybrid layer of ~20 micron thickness was detected irrespective of composite or dentine type (Figure 5c).

With acid etched dentine and no adhesive, the experimental material with 4-META could form a dentine/composite hybrid layer of ~10 micron. In addition, however, there could be some cracks visible within the compos[ite near this interface (Figure 5d). With no acid etching or adhesive and the 4-META composite gaps were <2 micron (Figure 5e). All other materials showed variable gaps of up to 20 micron between the composite and dentine when no adhesive was employed irrespective of acid treatment (Figure 5f). The SEM images in Figure 5g, and 5h shows that the weak point in composite-ibond-dentine complex is most of the time due to weakness at dentine interface, or with in the dentine itself (Figure 5b).

### 2.2. Discussion

#### 2.2.1. Chemical Properties of Dentine

The Raman spectra observed above for dentine and acid etched dentine were comparable with those observed previously for intact and carious dentine. Earlier studies additionally showed that etch depth was ~8 micron with 15 s etch of non-carious dentine but that this could be doubled if the dentine was carious [14]. The Raman microscope used in the above study provides the chemistry of the top 10–20 micron thick surface layer and is therefore ideal for assessing changes in chemistry upon acid etching. The relative levels of surface collagen and hydroxyapatite in the above new study suggests that chemically both 20 s etched human dentine and un-etched ivory are a good model for carious dentine. 20 s etched ivory would then be a model for etched carious dentine. The above new results also show acid clearly dissolves the carbonate associated with hydroxyapatite. Carbonate is formed by dissolution of carbon dioxide (e.g., from carbonated drinks) in saliva and is able to replace phosphate ions in hydroxyapatite [15]. This increases the solubility of the hydroxyapatite [16]. The lack of carbonate in ivory is consistent with lack of contact with food and saliva.

#### 2.2.2. Microscopic Properties of Dentine

Microscopic analysis showed that ivory tubules were of comparable size and shape to those in human dentine but of lower density. Sizes observed were comparable with those observed previously with human dentine [17,18,19]. Tubule density increasing in human dentine closer to the pulp has been previously reported [20].

#### 2.2.3. Mechanical Properties

The above bending strength of the human dentine is comparable with that observed in the literature (between 15 [21], and 200 MPa [22,23,24,25]). The broad range of values observed was a consequence of the large number of factors that affect this property. Strength has been shown to increase if the tubules are aligned parallel to the direction of applied force [25] or if the dentine is heated [26]. A reduction in tubule density would also enhance strength. Changes in chemistry, including hydroxyapatite and water content or collagen structure, might also modify strength. Collagen consists of rigid rod like triple helices with crosslinks that bind them into fibrils. Water can penetrate between the helices and expand the fibrils [27]. Hydroxyapatite precipitates around the fibrils [28] stabilizing the collagen against excessive water sorption induced expansion and attack by enzymes. The above new studies suggest that enhancing water content of the ivory has little effect on strength. This might be a consequence of the plasticizing effect of excessive water sorption being compensated by ionization of the collagen enhancing forces of attraction between the collagen fibrils. The higher strength of the human dentine compared with wet ivory could be a consequence of the higher hydroxyapatite content limiting water sorption and thereby compensating the higher density of tubules. On average the maximum amount of water absorbed by human and dry ivory dentine was ~10 w/w %, and 15 w/w % respectively [29].

Increasing water content of collagen can reduce its dry modulus from ~5 GPa down to a few MPa [30]. Increasing volume density of the tubules will decrease modulus but addition of rigid hydroxyapatite (modulus ~100 GPa ) enhances modulus [31]. The above observed reduction in modulus of the ivory upon hydration will be due to plasticization of the collagen. The higher modulus of human dentine compared with wet ivory could be primarily due to the increased hydroxyapatite.

From the above study it is clear that water sorption enhances the flexural strain for ivory but that it is even higher for the more mineralized human dentine. A possible explanation could be that in water, positive and negative charges may form (on e.g., lysine and aspartic acid amino acids respectively) in collagen providing inter-fibril interactions. The precipitation of hydroxyapatite could further enhance these ionic interactions. Stronger ionic attractions between the collagen fibrils would enable them to be pulled further apart before breaking [32].

#### 2.2.4. Adhesion Properties

Although there has been much criticism of the reproducibility of the shear bond test, it is still commonly used as a first choice for dentine adhesion studies [33]. From the above, the biggest factor increasing bond strength was use of the adhesive ibond. The values obtained for human dentine using ibond are in agreement with previous studies using un-etched human dentine [34]. In the presence of water the anhydride group in the 4-META within ibond is hydrolyzed to provide two carboxylic acid groups. It is proposed that these may partially demineralize the dentine to allow some micro-mechanical interlocking, but in addition enable a chemical bond with calcium in remaining hydroxyapatite. Furthermore, it may bond with basic amino acid groups in the collagen. The low viscosity as a result of the solvent in ibond also enables increased penetration into demineralized dentine. After solvent evaporation upon air drying, adhesive polymerization additionally provides strong chemical bonds with the monomers in the composite [35]. The ibond UDMA hydrophobicity aids intermixing with the composites. The above SEM images suggest that most often, bond failure occurs at the dentine interface or in the dentine itself rather than at any composite-ibond interface.

Upon acid etching of the human dentine, the above results showed the adhesive forms a much thicker interface layer. This, however, caused only a small increase in the average bond strength consistent with previous studies [36]. The interface layer thickness observed above was comparable to the depth of acid etching previously observed with phosphoric acid use [14]. This layer will consist largely of a mixture of residual hydroxyapatite crystallites and collagen fibrils with adhesive monomers replacing displaced unbound water [37]. The solvents, hydrophilic monomers and low viscosity, aid adhesive penetration into water filled collagen and tubules. Acid etching also enables greater penetration of adhesives into tubules which may potentially further enhance interlocking between the adhesive and dentine [38]. The lack of any significant difference in the bond strengths for ivory with lower density of tubules instead of human dentine, suggests this mechanism of bonding had limited additional benefit when ibond was employed.

The very low bond strength of the conventional composites (Z250, Gradia and C-HEMA) to un-etched human dentine will be due to lack of any mechanism for chemical or micromechanical bonding. The greater bond strengths obtained with Vertise flow is in agreement with previous studies [39]. This has been attributed to both lower viscosity as a result of lower filler content and the addition of glycerol phosphate dimethacrylate (GPDM) [40,41] which can form ionic bonds to calcium. The comparable bond strength for the higher viscosity C-4META composite suggests the ionic interactions may be more important in this case than filler loading.

The increased bond strength after etching of human dentine for the C-HEMA composite but lack of improvement upon using ivory could suggest that this composite may require penetration into tubules to improve bonding. This may have been improved by the addition of the low viscosity and hydrophilic HEMA [42]. With Gradia and Z250 improved bonding to etched ivory may be a consequence of these materials having some weak micro-mechanical interaction with highly demineralized collagen. Vertise flow may be able to bind un-etched ivory less well due to lack of hydroxyapatite. This problem may have been reduced with the C-4META composite by enhanced acidity [41] and therefore increased etching and chemical interaction.

The advantages of higher filler content in the 4-META composites compared with Vertise Flow include increased strength and lower shrinkage of the composite upon polymerization [43,44,45]. The SEM images show that when the bond strength is low the material can upon polymerization generate a gap between the dentine and composite. The size of the gaps observed were comparable with what might be expected from the known sample dimensions and composite shrinkages upon polymerization. These are typically ~3% for conventional composites with around 80 wt % filler [46] but 4.5% for Vertise flow with 70 wt % filler [47]. The higher filler content, in combination with moderate bond strengths would explain the limited gaps when C-4META was employed.

## 3. Experimental Section

### 3.1. Dentine Source

Elephant ivory dentine was used as a model substrate and was provided by the U.K Border Agency, Heathrow airport (CITES Reference 08/2012) for research purpose only. It should be noted that the authors do not advocate the use of ivory for any purposes and are thankful for CITES to allow the use of confiscated ivory that would be otherwise destroyed for academic research.

Human dentine was collected from non-carious adult human teeth. The teeth were collected through the UCL Eastman Bio bank after ethical approval and patient consent (Bio bank ID number 1304). The uncut teeth were stored in a 0.2% thymol solution at 4 °C for up to 4 weeks prior to use. The dentine was taken directly beneath the occlusal part of the enamel.

### 3.2. Composite Preparation/Source

The commercial dentine adhesive and etchant employed were ibond total etch (batch 010037) and ibond 35% phosphoric acid gel (batch 395074) (Heraeus Kulzer, Germany). ibond total etch consists primarily of the high molecular weight dental monomer urethane dimethacrylate (UDMA), adhesion promoting and demineralizing 4-methacryloxyethyl trimellitic anhydride (4-META), acetone and water [48]. The low viscosity of this adhesive enables better flow and penetration into rough dentine surfaces and tubules. Commercial composites examined included Z250 (3M™, Bracknell, UK, batch 202464, shade B3), Gradia Direct PA-2 (GC Corporation, Newport Pagnell, UK, batch 105936, shade P-A2) and Vertise flow (Kerr Dental Supply, Peterborough, UK, batch 1110402, shade A1). Z250 contains 82 wt % inorganic fillers. Gradia and Vertise flow contain 77 and 70 wt % fillers respectively some of which is pre-polymerized polymer. All these composites contain light curable high molecular weight hydrophobic dimethacrylate monomers. In addition, Vertise Flow contains glycerol phosphate dimethacrylate which has the potential to bond to hydroxyapatite. Their detailed composition has been provided elsewhere [49,50].

Experimental light curable dental composites were prepared with 80 wt % silanated barium alumino silicate glass with an average particle diameter of 7 μm (DMG, Hamburg, Germany). The monomer phase was formulated using UDMA and triethylene glycol dimethacrylate (TEGDMA) (DMG, Hamburg, Germany) in 3:1 weight ratio. 1 wt % camphorquinone (Polysciences, Warrington, PA, USA) and 1 wt % N, N-dimethyl-p-toluidine (Sigma Aldrich, UK) initiator and activator were added. Furthermore, 5 wt % of either the hydrophilic monomer 2-Hydroxy ethyl methacrylate (HEMA) (DMG, Hamburg, Germany) or 4-META (Polysciences, Warrington, PA, USA) were added. Formulations were mixed using a Speed Mixer™ DAC 150.1 FVZ (Synergy Devices Ltd, High Wycombe, UK) at room temperature for 20 s at 3000 rpm. The experimental composites had greater viscosity than Vertise flow but lower viscosity than Z250 and Gradia.

### 3.3. Raman Spectra of Dentine

Raman mapping spectroscopy (Horiba Jobin Yvon, Paris, France) was used to generate average Raman spectra of the surfaces of both dentine before and after treatment with 35% phosphoric acid gel for 20, 60, or 120 s. After etching, each sample was thoroughly washed with distilled water for about 30 s, and blotted dry.

Raman were obtained between 800 and 1800 cm^−1^ using a confocal hole of 150 μm, 632.8 nm He-Ne laser and 50x microscope objective. For each specimen, spectra were obtained by mapping areas of 40 × 40 μm. In each area 16 spectra were obtained for 20 s each. After baseline subtraction, spectra were normalized by the Amide I peak at 1670 cm^−1^ and then averaged. This was repeated 5 times for each treatment and dentine type using multiple sites. Average normalized intensities as a function of wavenumber and 95% confidence intervals were calculated. Intensity of the hydroxyapatite peaks at 961 cm^−1^ were plotted against the etchant time.

### 3.4. Flexural Properties of Dentine

For assessment of dentine strength, modulus, and strain, both dentine were cut into rectangular sections of 15 × 5 × 2 mm. The ivory samples were stored dry at room humidity/temperature or in distilled water for 24 h at room temperature. Cut human dentine specimens were all stored in 0.2% thymol at 4 °C before testing. Specimen thickness was checked using digital vernier calipers (Moore and Wright, West Yorkshire, UK). Strength was assessed using a “3-point bending” jig. This consisted of two support rollers 5.0 mm in diameter. The centers of support rollers were 10.0 mm apart. Load was applied at the midpoint, between the supports by means of a third roller 3 mm in diameter.

The load was applied with a 1 kN load cell, at a cross head speed of 1 mm/min using a computer-controlled universal testing machine (Instron 4502, Bucks, UK). The modulus *E*, 3-point bending strength σ, and strain ϵ were determined using Equations (2)–(4).

(2) $$E=\frac{\text{m}L^{3}}{4bh^{3}}\;$$

(3) $$\text{σ}=\frac{3FL}{2bh^{2}}$$

(4) $$\epsilon =\frac{6\text{Dh}}{L^{2}}$$

*F* is the maximum load on the load deflection curve, *L* is the length of support span, *b* is the width of tested specimen, *h* is the thickness of tested specimens, m is the gradient (*i.e.*, slope) of the initial straight-line portion of the load deflection curve, and D is the maximum displacement of the tested specimen from its original position to the point of highest load. Sample repetition was 15.

Data were fitted to a Weibull type expression:

*P_f_* = 1 − exp (−σ*/*σ_θ_ )*^m^* (5)

*m*–Weibull shape parameter, σ–bending strength, (which can be replaced by modulus, or flexural strain) of each specimen and σ_θ_–Weibull scale parameter. When σ *=* σ_θ_, *P_f_* = 63.2%. *P_f_* was defined as (*i* − 0.5)/*n*, where *n* is the number of specimens and *i* is the rank of a specimen in a list when strength, modulus or strain are ordered from lowest to highest values. Rearranging and taking double logs of Equation (5) gives:

lnln [1/(1 − *P_f_*)] = *m* ln σ − *m* ln σ_θ_ (6)

Weibull parameters were calculated from the slope and intercept of the left hand side of Equation (6) plotted *vs.* ln σ [51].

### 3.5. Shear Bond Strength of Composites to Dentine

Cut ivory dentine cubes (~1 × 1 × 1 cm) obtained from middle of a single tusk were placed in water for 24 h at 37 °C. Thereafter they were kept in small sealed containers to reduce water evaporation and used within 48 h. Fifteen human teeth were used in shear bond test. Both dentine cubes, and 0.2% thymol stored human teeth were cut vertically in half, and embedded in slow-setting viscous self-curing resin such that dentine tubules were perpendicular to the top resin surface. P120 paper was used to grind the resin surface until sufficient dentine was exposed. The dentine was then polished with P500 paper until the surface was even and smooth when visually inspected.

To assess bond strength, composite pastes were poured in 2 mm increments into a brass tube of 3 mm internal diameter, and 6 mm long placed on the surface of the dentine. The end of the tube in contact with dentine was chamfered at 45 degrees to reduce its contact area. Each 2 mm increment was cured for 40 s. Immediately before application of the composite, the dentine was pretreated by

(1) ibond application and light cure for 20 s as per manufacturer’s instructions; or
(2) acid etch application for 20 s followed by water rinsing, gentle drying and ibond application and cure; or
(3) acid etch for 20 s, rinse and dry; or
(4) no acid or ibond treatment.

The shear bond test was done according to ISO standard [52]. Shear bond strength was determined using an Instron Universal testing machine with a “Flat-edge shear fixture” jig. The jig consisted of a metal holder with an adjustable screw to secure the specimen and an adjustable blade, which was used to shear the tube from the dentine. A 1 kN load cell at cross head speed of 1 mm/min was used.

The load at break was recorded and the bond strength *τ* calculated using Equation (7), in which *F* is the load at break, and *A* is the bonded area of the cylinder.

(7) $$\text{τ}=\frac{F}{A}\;$$

Each specimen was repeated 8 times, and 95% confidence interval was calculated from standard deviation using Equation (8).

(8) $$CI=\frac{2SD}{\sqrt{n}}$$

### 3.6. Scanning Electron Microscopy

To visually assess micro-gap formation due to composite shrinkage, dentine blocks of 5 mm depth were produced containing cavities of 3 mm diameter. After drilling, the cavities were washed and then treated in the four different dentine pre-treatment methods above. Following treatment the cavities were restored by placing composites in 2 mm layers and curing as above. After 24 h in distilled water at room temperature, restored cavities were cut in half vertically to expose the interface on two sides and from top to bottom of the restoration. The surface of both dentine were polished using P2400 paper and sputter coated with gold/palladium before taking microscopic images of interfaces using a Scanning electron microscope (SEM)(Phillip XL-30, Eindhoven, The Netherlands).

## 4. Conclusions

In this study, dentine from different sources were analyzed. The ratio of hydroxyapatite to collagen in ivory was half that in human dentine and comparable to that of human dentine etched for 20 s with acid. Surface ivory and human dentine hydroxyapatite level decreased by half with every 23 s of acid etch. The bending strength of ivory was approximately half that of human dentine presumably due to lower hydroxyapatite content.

Additionally, to characterization of dentine the experimental and commercial composites bond strengths and micro-gap formation were investigated. The major factor increasing composite bond strength to dentine was the use of ibond. Acid etching and the addition of acidic monomers in the composites could increase bond strength in some circumstances.
